# Depressive symptoms and axial motor disorders in individuals with Parkinson's disease: a cross-sectional study

**DOI:** 10.1055/s-0042-1758444

**Published:** 2022-12-28

**Authors:** Nathalie Ribeiro Artigas, Ana Carolina Leonardi Dutra, Nayron Medeiros Soares, Gabriela Magalhães Pereira, Vanessa Bielefeldt Leotti, Julia Schneider Krimberg, Aline de Souza Pagnussat, Carlos Roberto de Mello Rieder

**Affiliations:** 1 Universidade Federal do Rio Grande do Sul, Programa de Pós-Graduação em Ciências Médicas, Porto Alegre RS, Brazil.; 2 Universidade Federal do Rio Grande do Sul, Programa de Pós-Graduação em Epidemiologia, Departamento de Estatística, Porto Alegre RS, Brazil.; 3 Pontifícia Universidade Católica do Rio Grande do Sul, Escola de Ciências da Vida e da Saúde, Porto Alegre RS, Brazil.; 4 Programa de Pós-Graduação em Ciências da Reabilitação, Universidade Federal de Ciências da Saúde de Porto Alegre, Porto Alegre RS, Brazil.; 5 Universidade Federal de Ciências da Saúde de Porto Alegre, Departamento de Clínica Médica, Divisão de Neurologia, Porto Alegre RS, Brazil.

**Keywords:** Depression, Posture, Postural Balance, Photogrammetry, Muscle Rigidity, Depressão, Postura, Equilíbrio Postural, Fotogrametria, Rigidez Muscular

## Abstract

**Background** Depression is an important nonmotor symptom of Parkinson's disease (PD) and has been associated with the motor symptoms in these individuals.

**Objectives** To determine whether there are relationships between depressive symptoms and abnormalities in axial postural alignment and axial motor deficits, especially postural instability, and trunk rigidity in PD.

**Methods** In this cross-sectional study, 65 individuals were evaluated using the Beck Depression Inventory-II (BDI-II) for the analysis of depressive symptoms and underwent a postural assessment of head, trunk, and hip sagittal alignment through computerized photogrammetry. The MDS-UPDRS was used to assess clinical aspects of PD, the Trunk Mobility Scale was used to assess axial rigidity, and the MiniBESTest to assess balance. To determine the relationship between depressive symptoms and postural alignment, multiple linear regression analysis was performed.

**Results** The participants with depressive symptoms had more severe motor deficits as well as greater trunk rigidity and worse postural instability ( *p* < 0.05). When the postural angles were compared between men and women using Student's *t* -test, it was found that men had greater flexion angles of the head ( *p* = 0.003) and trunk ( *p* = 0.017). Using multiple linear regression analysis corrected for the age and sex of the participants, we verified that the anterior trunk inclination was significantly larger in the PD population with depressive symptoms (R ^2^ = 0.453, β = 0.116, and *p* = 0.045).

**Conclusion** PD individuals with depressive symptoms have more severe flexed trunk posture, mainly in older men. Additionally, more severe depressive symptoms are associated with worsening postural instability, trunk rigidity and motor deficits in this population.

## Resumo

**Antecedentes** A depressão é um sintoma não motor importante da doença de Parkinson (DP) e tem sido associada aos sintomas motores nesses indivíduos.

**Objetivos** Determinar se existem relações entre sintomas depressivos e anormalidades no alinhamento postural axial e déficits motores axiais, especialmente instabilidade postural e rigidez de tronco na DP.

**Métodos** Neste estudo transversal, 65 indivíduos foram avaliados pelo BDI-II para análise de sintomas depressivos e submetidos à avaliação postural do alinhamento sagital de cabeça, tronco e quadril por meio de fotogrametria computadorizada. A MDS-UPDRS avaliou os aspectos clínicos, TMS avaliou rigidez axial e o MiniBESTest equilíbrio. Para determinar a relação entre sintomas depressivos e alinhamento postural, realizou-se uma análise de regressão linear múltipla.

**Resultados** Os participantes com sintomas depressivos apresentaram déficits motores mais graves, bem como maior rigidez de tronco e pior instabilidade postural ( *p* < 0,05). Quando comparados os ângulos posturais entre homens e mulheres pelo teste *t* de Student, verificou-se que os homens apresentaram maiores graus de flexão da cabeça ( *p* = 0,003) e do tronco ( *p* = 0,017). Por meio da análise de regressão linear múltipla corrigida para a idade e sexo dos participantes, verificamos que a inclinação anterior do tronco foi significativamente maior nos indivíduos com DP com sintomas depressivos do que sem sintomas depressivos (R ^2^ = 0,453, β = 0,116 e *p* = 0,045)

**Conclusão** Indivíduos com DP com sintomas depressivos apresentam postura de tronco flexionado mais severa, principalmente em homens mais idosos. Além disso, os sintomas depressivos mais graves pioram significativamente a instabilidade postural, a rigidez do tronco e os déficits motores nessa população.

## INTRODUCTION

Parkinson disease (PD) is a progressive neurodegenerative condition characterized by movement disorders such as bradykinesia, rigidity, resting tremor and postural instability, 1 and it is accompanied by nonmotor symptoms such as psychiatric, cognitive, gastrointestinal and autonomic symptoms. 2 3 4 Depressive symptoms in particular are common in individuals with PD, and the proportion of depression diagnosis is four times higher among PD patients than among the general population. 5 Furthermore, depressive symptoms have previously been associated with the worsening of cardinal motor symptoms, especially in patients with a predominance of rigid akinetic symptoms. 6

Studies conducted with subjects with and without depressive disorder claim that a stooped posture may be considered a typical feature in patients with depression. 7 8 Posture can be assessed using computational methods such as computerized photogrammetry. This kind of assessment is an effective and safe method for evaluating, analyzing, and quantifying postural abnormalities (PAs). 9 10

Nevertheless, the influences of depression and depressive symptoms on axial parameters and functionality when a neurodegenerative condition such as PD is also present are unclear. The study by Kim et al., 11 for example, reports that the curvature of the pelvis can be a marker of depression in PD, but it does not present sufficient results to confirm this relationship between the axial posture and depressive symptoms, or their relationship with other motor aspects of PD.

We hypothesized that depressive symptoms in PD impact the severity of PAs and axial motor deficits. Therefore, the primary purpose of this study was to identify the relation between depressive symptoms and severity of axial motor disorders in individuals with PD. The secondary objectives were to investigate the associations between depressive symptoms and axial PA severity and the contribution of depressive symptoms to motor disorders of this population.

## METHODS

This is a cross-sectional study with quantitative data analysis. The study population consisted of individuals who had a clinical diagnosis of PD, as defined by the London Brain Bank criteria, 12 that corresponded to a severity between stages 1 and 4 according to the Hoehn & Yahr staging scale (H&Y) 13 and were able to remain standing in the upright position for at least 10 seconds. Most participants were patients in the Movement Disorders Outpatient Clinic at the Hospital de Clínicas de Porto Alegre (HCPA). Individuals who had limitations due to orthopedic, rheumatological, or other diagnosed neurological diseases; were using deep brain stimulation; or were diagnosed with dementia were excluded from the study.

### Procedures and instruments for data collection

After the research ethics committee of the HCPA (CAAE number 67433517.5.0000.5327) approved the study, patients from the neurology unit in the hospital were invited to participate. All subjects provided signed consent before the evaluations were initiated, and those who met the inclusion criteria were included in the study population.

The assessment began with a general anamnesis to verify the patient's sociodemographic and clinical history. Next, a postural evaluation was performed using a computerized photogrammetry protocol with the Postural Assessment Software (PAS/SAPO, BMClab – UFABC, São Paulo, Brazil). 14 15 A non-zoomed Sony H Series Dsc-h300 20.1mp (Sony Corp., Minato, Tokyo, Japan) camera was used to capture images of the subject in accordance with the protocol recommended by the SAPO software, 15 with a plumb line attached to the ceiling and two small balls spaced one meter apart and glued to the wire for image calibration. To ensure high photo quality, well-configured and calibrated photography equipment were used, the evaluation was performed on level ground and in a comfortable space and temperature, the privacy of the participant was respected, and adequate lighting was provided to enable a precise focus. 16 The participants were positioned beside the plumb line and perpendicular to the axis of the digital camera, which was located 2.3 m away and supported on a one-meter high tripod.

Anatomical references, which served as guides for the image analysis, were marked with 15 mm reflective styrofoam spheres placed at eight points on the right and left sides of each participant: 15 1–tragus; 2–acromium; 3–greater trochanter; and 4–lateral malleolus. After the reflective markers were placed, the subjects were asked to stand facing forward in a relaxed manner. To ensure that the subjects were within 10 cm of the wall and plumb line, a black mat was placed at the location where they should be positioned standing upright. The participants were given verbal commands to assume a comfortable and habitual position, with their feet in a self-selected position. 17 All participants were evaluated at the “on” state and were photographed in the left and right profile postures. The anatomical markers were placed, and the images were taken by a trained evaluator.

**Figure 1:**
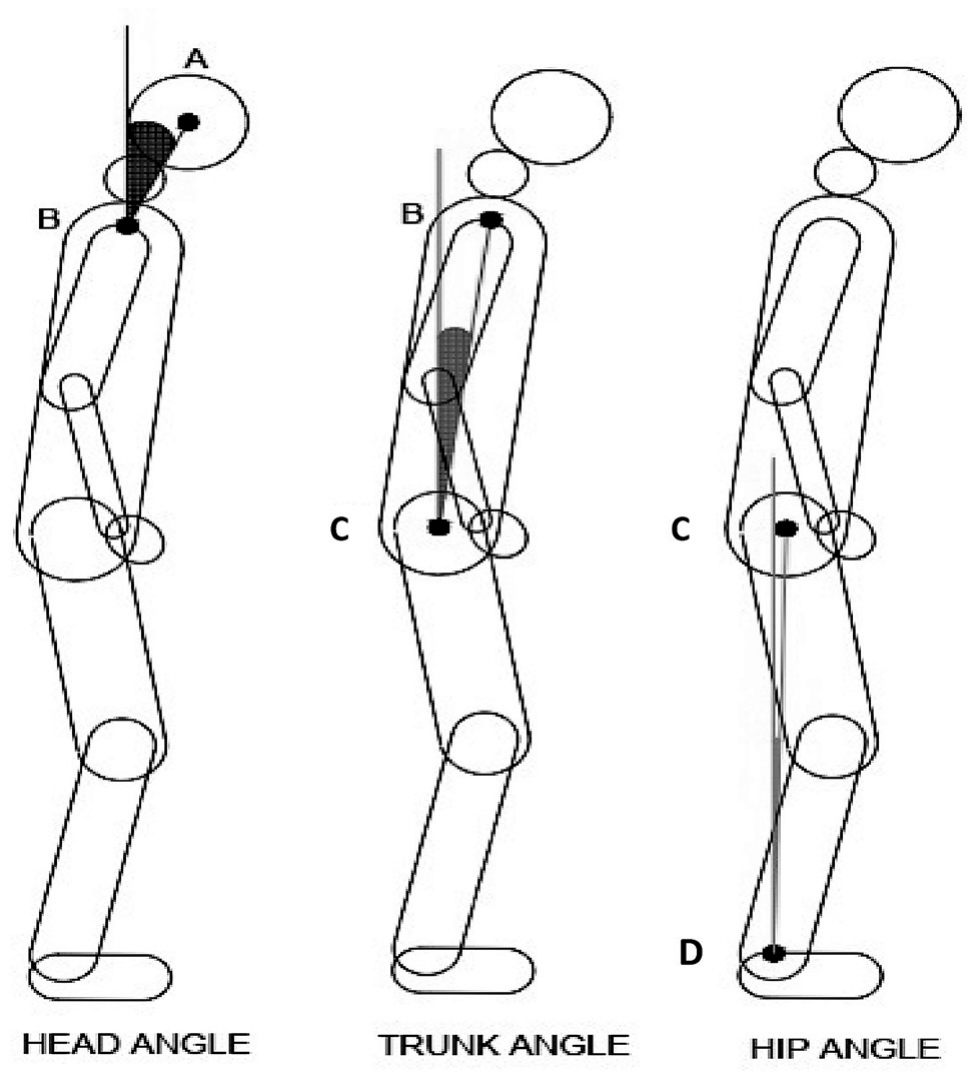
Angles and anatomical points used for analysis of axial postural alignment in sagittal plane.

After the images were acquired, they were analyzed by a single blinded researcher using the ImageJ (LOCI, University of Wisconsin, Madison, WI, USA) software. For the analysis of the vertical alignment of the head, we used the angle formed by the tragus, acromion, and a vertical line; for the trunk alignment, we used the angle formed by the acromion, greater femoral trochanter, and a vertical line; and for the hip angle, the anatomical point of the greater trochanter of the femur, lateral malleolus, and a vertical line were used. 15 All angles that were analyzed are illustrated in Figure 1 .

Depressive symptoms were assessed using the Beck Depression Inventory-II (BDI-II). 18 The subjects were later divided into two groups, based on the final scores: the “with depressive symptoms group” (BDI-II ≥ 14) and “without depressive symptoms group” (BDI-II < 14), as suggested by Schrag et al. 16 They were also evaluated using the Portuguese language version of the Movement Disorder Society Unified Parkinson Disease Rating Scale (MDS-UPDRS) 19 to verify the clinical aspects of PD; the H&Y scale 13 for disease staging; the Trunk Mobility Scale (TMS) for the axial rigidity evaluation, 20 and a short version of the Balance Evaluation Systems Test (MiniBESTest) for the balance assessment. 21

The participants were also classified according to their motor subtype into the tremor dominant group (TD), postural instability/gait difficulty (PIGD), or indeterminate type (IT) using the MDS-UPDRS scores. 22 The axial subscore was calculated from the sum of items 1 and 9–13 of part III of the MDS-UPDRS, as suggested by Li et al., 23 and the entitled posture subscore is equivalent to the score obtained in item 3.13 of the MDS-UPDRS.

Except for the MiniBESTest, in which higher scores corresponded to better balance (maximum score indicates normality), in all the scales used, a higher score corresponded to more severe symptoms.

### Statistical data analysis

The qualitative characteristics were described by frequencies and percentages, while the quantitative characteristics were described by means and standard errors (SE), or medians and interquartile ranges (IQRs).

The joint angles were measured in degrees, with a positive sign being adopted for anterior inclination angles (flexion joint), and a negative sign for posterior inclination angles (extension joint). For the final values, we used the average of the angles obtained in the photos of the right and left sagittal profiles of each participant.

The normality of the angles calculated by photometry was verified by the Shapiro-Wilk test and normal probability graphs. Differences between the depressed and nondepressed groups were evaluated with the chi-square or Fisher exact tests for the qualitative characteristics, and with the Mann-Whitney or Student *t* -tests for the quantitative characteristics. The relationships between the BDI-II scores and angles or other clinical outcomes were evaluated using the Spearman correlation coefficient and Pearson correlation coefficient for the variable levodopa equivalent dose (LED). To determine the relation between the BDI-II scores and evaluated joint angles, multiple linear regression analysis was performed with the participant's age, diagnostic time, total MDS-UPDRS score, LED, and sex included as covariates, considering they have been described in the literature as factors that interfere with motor aspects and PD postures. 24 25 The analysis was performed using the PASW v18.0 software (SPSS Inc., Chicago). The adopted significance level was 5%.

## RESULTS

A total of 79 participants were included in this study. However, 14 of them were unable to complete all stages of the clinical evaluation and were excluded: 4 because they could not remain standing while the images were taken, 3 because they had large dyskinetic movements when the images were taken, and 7 because they presented with severe cognitive deficits and were unable to comprehend the scales. Thus, the analyses were performed with a total of 65 individuals, of which 35 (53.8%) were men and 30 (46.2%) were women, aged between 40 and 79 years (mean = 62.59, SE = 1.22 years). Of the total number of participants, 33 (50.8%) used some type of antidepressant medication.

However, in the assessment of balance through the MiniBESTest, 9 participants failed to complete all the necessary activities for this assessment. Therefore, a total of 56 participants were included in the analysis of this specific variable.

Table 1 presents the clinical and motor differences between the groups of participants with depressive symptoms ( *n* = 34) and without ( *n* = 31). The groups were homogeneous regarding the age, diagnostic time, and sex of the participants. Additionally, both groups did not differ in terms of the use of antidepressant drugs and LED.

**Table 1:** Demographic and clinical characteristics of patients with Parkinson disease with and without depressive symptoms

Variables		With depressive symptoms		Without depressive symptoms		*p-* value
		( *n* = 34)	SE or % or IQR	( *n* = 31)	SE or % or IQR	
Age (years) ^a^		61.71	1.95	63.55	1.44	0.459 ^f^
Diagnostic time (years) ^a^		10.18	0.95	9.06	0.77	0.367 ^f^
Sex ^b^	Male	16	47.1	19	61.3	0.321 ^g^
	Female	18	52.9	12	38.7	
Hoehn & Yahr ^b^	Stage 1	3	8.8	7	22.6	0.026* ^e^
	Stage 2	16	47.1	20	64.5	
	Stage 3	11	32.4	4	12.9	
	Stage 4	4	11.8	0	0	
Motor subtypes ^b^	TD	9	26.5	16	51.6	0.117 ^g^
	PIGD	20	58.8	13	41.9	
	TI	5	14.7	2	6.5	
MDS-UPDRS Score Part I ^a^		19.82	1.42	9.68	0.97	0.000* ^f^
MDS-UPDRS Score Part II ^a^		20.53	1.4	14.97	1.47	0.008* ^f^
MDS-UPDRS Score Part III ^a^		47.0	2.11	41.10	2.44	0.072 ^f^
MDS-UPDRS Score Part IV ^a^		7.26	0.98	5.16	1.03	0.147 ^f^
Total MDS-UPDRS Score ^a^		94.62	7.17	70.90	4.71	0.000* ^f^
Sum of axial subscore MDS-UPDRS ^c^		6.0	14.0	4.0	5.0	0.033*
Posture subscore MDS-UPDRS ^c^		1.0	1.0	1.0	1.0	0.356
MiniBESTest ( *n* = 56) ^c^		24.0	7.5	28.00	5.75	0.007* ^d^
TMS ^a^		9.65	0.55	7.71	0.61	0.022* ^f^
Head anteriorization angle (°) ^c^		23.2	12.67	23.01	11.6	0.948 ^f^
Trunk flexion angle (°) ^c^		4.38	4.89	2.91	4.44	0.212 ^f^
Hip flexion angle (°) ^c^		3.7	4.65	2.65	4.29	0.351 ^f^
Number of patients on antidepressant medication ^b^		21	61.8	12	38.7	0.084 ^g^
Total LED (mg/day) ^a^		1,276.27	108.22	1,015.34	72.39	0.054 ^f^

**Abbreviations:** IQR, interquartile range; LED, levodopa equivalent dose; MDS-UPDRS, unified Parkinson disease rating scale; SE, standard error; TMS, trunk mobility scale. **Notes:** ^a^ Variables described in mean (SE). ^b^ Variables described in N (%). ^c^ Variables described in median (IQR). ^d^ Mann-Whitney U test. ^e^ Fisher exact test. ^f^ Student *t* -test. ^g^ Chi-square test. * *p* ≤ 0.05.

In Table 1 , it can still be seen that the patients with depressive symptoms had a significantly higher disease severity according to the H&Y scale ( *p* = 0.026) and, when comparing the scores obtained in the MDS-UPDRS, the individuals in the group with depressive symptoms had significantly greater deficits in parts I and II, in the axial items, and in the total MDS-UPDRS score ( *p* < 0.005 in all these analyses).

Table 2 presents the Spearman correlation coefficients for the associations between the clinical variables and severity of depressive symptoms in patients with PD. The median BDI-II score was 13 points (IQR = 11 points) for the men and 16.5 points (IQR = 14 points) for the women. However, when the same data were compared using the Mann-Whitney U-test, the differences did not appear to be significant ( *p* = 0.333).

**Table 2:** Correlation coefficients for the associations between clinical variables and severity of depressive symptoms measured by BDI-II in patients with Parkinson disease

Variables	r	*p-* value
Age	-0.009	0.945
Diagnostic time	0.121	0.338
Hoehn & Yahr	0.402	0.001*
MDS-UPDRS Score Part I	0.658	0.0*
MDS-UPDRS Score Part II	0.389	0.001*
MDS-UPDRS Score Part III	0.344	0.005*
MDS-UPDRS Score Part IV	0.24	0.054
Total MDS-UPDRS Score	0.516	0.0*
Sum of axial subscore MDS-UPDRS	0.397	0.001*
Posture subscore MDS-UPDRS	0.16	0.203
MiniBESTest (n- = 56)	-0.366	0.006*
TMS	0.402	0.001*
Head anteriorization angle (°)	0.088	0.487
Trunk flexion angle (°)	0.203	0.105
Hip flexion angle (°)	0.193	0.123
Total LED (mg/day)	0.314 ^#^	0.011*

**Abbreviations:** BDI-II, Beck depression inventory; LED, levodopa equivalent dose; MDS-UPDRS, unified Parkinson disease rating scale; r, Spearman correlation coefficients; TMS, trunk mobility scale. **Notes:** ^#^ Pearson correlation coefficients. * *p* ≤ 0.05.

Through the postural evaluation with the photos, it was possible to verify that the men presented a mean anterior head inclination angle of 27.11° (SE = 1.93°), trunk flexion angle of 4.96° (SE = 0.87°), and hip flexion angle of 3.66° (SE = 0.67°). The women presented average angles of 18.44° (SE = 2.05°), 2.19° (SE = 0.67°), and 2.65° (SE = 0.91), respectively. These angles were compared between men and women using the Student *t* -test, and the results showed that the PA in the head ( *p* = 0.003) and trunk ( *p* = 0.017) were significantly worse in the group of male patients. However, the hip flexion angle did not differ significantly between sexes ( *p* = 0.369). Additionally, no participant had postural changes classified as camptocormia or Pisa syndrome.

Table 3 presents the results of the multiple regression analyses performed when using age, diagnostic time, motor subtype, and sex as covariates. These analyzes showed a predictor effect of 19.8% for severe depressive symptoms on larger anterior trunk inclination angles (β = 0.113 and *p* = 0.039), when the model was corrected for the participants' age and sex. Statistical analyzes were repeated in these models, adding the use of antidepressants and total MDS-UPDRS scores as predictors, and it was not significant in any of the two new outcomes.

**Table 3:** Multivariate regression of depressive symptoms and axial postural abnormalities in sagittal plane

Variables	R ^2^	β	*p-* value
Head anteriorization angle	0.323	0.138	0.308
Trunk flexion angle	0.453	0.116	0.045*
Hip flexion angle	0.167	0.110	0.052

**Notes:** Multiple linear regression using age, diagnostic time, motor subtype, and sex as covariates to analyze the relationship between depression and axial postural changes. R ^2^ coefficient of determination. β regression coefficient for BDI-II. * *p* ≤ 0.005.

**Figure 2:**
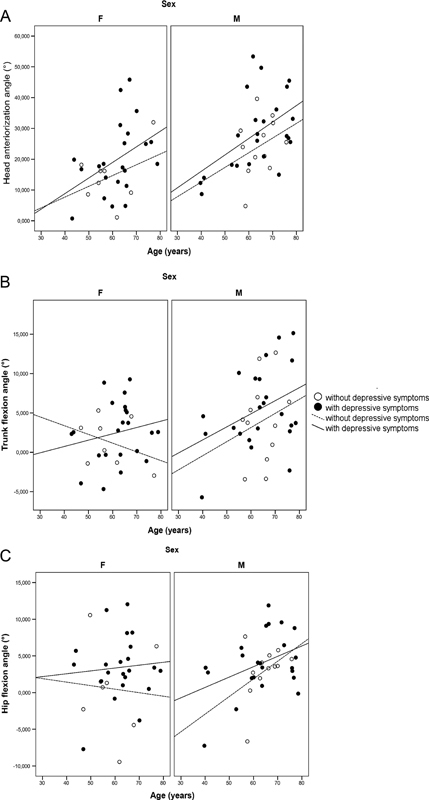
Distribution of individuals with and without depressive symptoms by age, gender, and degree of head anteriorization angle (A), trunk flexion angle (B), and hip flexion angle (C).

The distributions of individuals with and without depressive symptoms by age, sex, and degrees of anterior trunk flexion are illustrated in Figure 2 . Regression models using predictors of sex, age, and presence or absence of depressive symptoms were also fitted. No predictor showed statistical significance for hip postural changes. Age showed a significant positive relationship for the angles of head and trunk flexion (coefficients 0.515 and *p* < 0.001; 0.117 and *p* = 0.039, respectively). Women had a smaller angle on average for head and trunk (differences in relation to men of -8.6, *p* = 0.003, and -2.7, *p* = 0.017), showing that for the same age and the same group of depressive symptoms, men had more severe postural changes.

## DISCUSSION

The aim of this study was to verify the correlation between depressive symptoms and disturbance severity in axial posture, balance, trunk mobility, and motor and sociodemographic aspects in subjects with PD. It is noteworthy that the objective of the present study was not to diagnose depression, but to verify the existence and severity of depressive symptoms in individuals with PD who were part of our sample. Therefore, we separated them into groups with and without depressive symptoms.

In this sample, it was possible to verify that there is a predictive effect of the severity of depressive symptoms for greater angles of anterior trunk inclination, according to multiple regression analysis performed using age, diagnostic time, motor subtype, and sex as covariates. We also verified that men showed larger alterations in head and trunk alignment than women.

Additionally, the subjects with more depressive symptoms exhibited more serious motor deficits as well as greater trunk rigidity (worst results in the sum of MDS-UPDRS axial items and worst scores in the trunk mobility scale) and worse postural instability (MiniBESTest score). We also found a correlation between the severity of depressive symptoms and axial motor disorders.

Some studies have reported there is a relation between depression and PAs in individuals without PD; for example, shoulder protrusion was found in individuals with depressive symptoms, 26 and excessive trunk flexion was found in older women with more severe depressive symptoms. 27 Canales et al. 7 reported that patients with depression have larger degrees of head flexion and thoracic kyphosis compared with healthy people, and that this postural misalignment improves during the remission of depressive symptoms. This result has also been reported in patients with PD by Kim et al., 11 who found significant correlations between the severity of depression and PAs in the pelvic region in 46 PD patients, who were divided into groups of patients with and without depression. In the present study, we found a statistically significant positive correlation between the severity of depressive symptoms and a larger anterior trunk inclination angle.

We included age and sex as the covariates for this analysis because previous studies have confirmed that postural and depressive symptoms worsen with advancing age, and that there is a difference in this rate of progression between men and women. For example, Gong et al. 28 reported changes in parameters that describe body posture throughout aging and emphasized that for an individualized functional analysis, it is essential to consider age. Furthermore, in our study, the male participants had significantly larger head and trunk flexion angles than the women. This result can be explained by a milder deterioration of motor function and a slower striatal degeneration in women than in men, suggesting a more benign phenotype in women. Disease progression may be slowed in females by high levels striatal dopamine, possibly due to the activity of estrogens, as indicated by a single-photon emission computerized tomography (SPECT) imaging study. 29 30

As we found a correlation between the severity of depressive symptoms and motor symptoms, especially axial stiffness and postural instability, in people with PD, we suggest that psychosocial mechanisms may play an important role in axial changes in this population. Bartolic et al. 31 reported that axial stiffness is a probable cause of PAs, as the participants in their study showed an improved trunk posture after their neck stiffness was reduced with the administration of apomorphine. Papapetropoulos et al. 32 evaluated the UPDRS scores of patients with PD and demonstrated that the bradykinesia and axial rigidity scores are also higher in patients with depressive symptoms than in those without. Other studies have evaluated the presence of postural instability in PD patients with symptoms of depression and found that postural instability is significantly correlated with depressive symptoms in patients with PD, with is consistent with our results. 33 34

All these findings corroborate the hypothesis that emotions and bodily factors interact reciprocally as a form of nonverbal expression of emotion. 35 Some theories suggest that the reciprocal relationship between postural variations and the emotional states of individuals should be investigated, as the emotional experience affects somatovisceral and motor systems, or vice versa. 36 These compensations are likely to occur most clearly in individuals who have difficulty or cannot express their feelings using facial gestures, 37 much like PD patients, who often experience facial hypomimia with disease progression. For these individuals, posture becomes a means of expressing their feelings.

Specifically, in individuals with PD, the presence of an association of axial motor deficits with depressive symptoms raises the possibility that a shared underpinning pathophysiology is involved. Depression is well characterized anatomically and involves prefrontal cortex and cingulate deficits. 38 The neuroanatomical basis for postural instability and axial deficits is less clear. However, depression and postural instability all localize to the basal ganglia circuitry, share dopaminergic dysfunction, and are generally considered levodopa-resistant entities. 39

A limitation of this study is that most participants had mild to moderate disease severity, so our results cannot be generalized to those with more advanced stages of PD. Moreover, absence of a control group with healthy individuals is a methodological weakness. Finally, it was not possible to identify the causal relationship between the depressive symptoms and posture, or whether they are influenced by the progression of PD symptoms. Therefore, prospective studies should be conducted to further investigate how depression and posture are related, and how PD affects them.

This is a cross-sectional study that verifies the association between the variables studied, and to establish a cause-effect relationship, we suggest carrying out a cohort study. Future research will be required to better define the pathophysiology of depression, PAs, and axial motor deficits. Addressing these issues in PD patients is fundamental, as they are factors that influence their functionality and quality of life. We suggest that additional studies are conducted to explore depressive symptoms, their relationships with other nonmotor symptoms and the relationships of PAs with other psychosocial symptoms, such as cognition and quality of life. Additionally, it is of great importance to verify the effectiveness of potentially effective treatments for depression in improving motor symptoms and PAs in this population.

Therefore, we conclude that depressive symptoms are nonmotor symptoms of PD that correlate with the severity of a flexed truncal posture, especially in male and older populations. Furthermore, more severe depressive symptoms are associated with worsening postural instability and trunk rigidity. Individuals with PD who present with depressive symptoms also have more disabling motor deficits than those without depressive symptoms. Finally, we suggest a future cohort study to define a cause-effect relationship between depressive symptoms and postural changes in this population.

## References

[1] Poewe W Seppi K Tanner C M Parkinson disease Nat Rev Dis Primers 2017 3 17013 28332488 10.1038/nrdp.2017.13

[2] National Institute for Clinical Excellence Chaudhuri K R Healy D G Schapira A H Non-motor symptoms of Parkinson's disease: diagnosis and management Lancet Neurol 2006 5 03 235 245 16488379 10.1016/S1474-4422(06)70373-8

[3] Hsu Y T Liao C C Chang S N Increased Risk of Depression in Patients with Parkinson Disease: A Nationwide Cohort Study Am J Geriatr Psychiatry 2015 23 09 934 940 25529799 10.1016/j.jagp.2014.10.011

[4] Rodriguez-Oroz M C Jahanshahi M Krack P Initial clinical manifestations of Parkinson's disease: features and pathophysiological mechanisms Lancet Neurol 2009 8 12 1128 1139 19909911 10.1016/S1474-4422(09)70293-5

[5] Veiga B A Borges V Silva S M Goulart FdeO Cendoroglo M S Ferraz H B Depression in Parkinson's disease: clinical-epidemiological correlates and comparison with a controlled group of non-parkinsonian geriatric patients Rev Bras Psiquiatr 2009 31 01 39 42 19506774 10.1590/s1516-44462009000100010

[6] Reijnders J SAM Ehrt U Lousberg R Aarsland D Leentjens A F The association between motor subtypes and psychopathology in Parkinson's disease Parkinsonism Relat Disord 2009 15 05 379 382 18977165 10.1016/j.parkreldis.2008.09.003

[7] Canales J Z Cordás T A Fiquer J T Cavalcante A F Moreno R A Posture and body image in individuals with major depressive disorder: a controlled study Rev Bras Psiquiatr 2010 32 04 375 380 21308258 10.1590/s1516-44462010000400010

[8] Canales J Z Fiquer J T Campos R N Soeiro-de-Souza M G Moreno R A Investigation of associations between recurrence of major depressive disorder and spinal posture alignment: A quantitative cross-sectional study Gait Posture 2017 52 258 264 27987469 10.1016/j.gaitpost.2016.12.011

[9] Braz R G Goes F PDC Carvalho G A Confiabilidade e validade de medidas angulares por meio do software para avaliação postural Fisioterapia em Movimento 2008 21 03 117 126

[10] Iunes D Castro F A Salgado H S Confiabilidade intra e interexaminadores e repetibilidade da avaliação postural pela fotogrametria Rev Bras Fisioter. 2005 9 327 334

[11] Kim Y Cheon S M Youm C Son M Kim J W Depression and posture in patients with Parkinson's disease Gait Posture 2018 61 81 85 29306811 10.1016/j.gaitpost.2017.12.026

[12] Calne D B Snow B J Lee C Criteria for diagnosing Parkinson's disease Ann Neurol 1992 32(Suppl):S125 S127 1510370 10.1002/ana.410320721

[13] Hoehn M M Yahr M D Parkinsonism: onset, progression and mortality Neurology 1967 17 05 427 442 6067254 10.1212/wnl.17.5.427

[14] Ferreira E AG Postura e controle postural: desenvolvimento e aplicação de método quantitativo de avaliação posturalSão Paulo Fac. Med. Univ. São Paulo.2005 144

[15] Ferreira E AG Duarte M Maldonado E P Burke T N Marques A P Postural assessment software (PAS/SAPO): Validation and reliabiliy Clinics (São Paulo) 2010 65 07 675 681 20668624 10.1590/S1807-59322010000700005 PMC2910855

[16] Schrag A Barone P Brown R G Depression rating scales in Parkinson's disease: critique and recommendations Mov Disord 2007 22 08 1077 1092 17394234 10.1002/mds.21333 PMC2040268

[17] Watson A WS Procedure for the production of highquality photographs suitable for the recording and evaluation of postureRev Fisioter Univ São Paulo.1998 5

[18] Gomes-Oliveira M H Gorenstein C Lotufo Neto F Andrade L H Wang Y P Validation of the Brazilian Portuguese version of the Beck Depression Inventory-II in a community sample Br J Psychiatry 2012 34 04 389 394 10.1016/j.rbp.2012.03.005 23429809

[19] Movement Disorder Society UPDRS Revision Task Force Goetz C G Tilley B C Shaftman S R Movement Disorder Society-sponsored revision of the Unified Parkinson's Disease Rating Scale (MDS-UPDRS): scale presentation and clinimetric testing results Mov Disord 2008 23 15 2129 2170 19025984 10.1002/mds.22340

[20] Franco C RC Leão P Townsend R Rieder C R Reliability and validity of a scale for measurement of trunk mobility in Parkinson's disease: Trunk Mobility Scale Arq Neuropsiquiatr 2011 69 04 636 641 21877033 10.1590/s0004-282x2011000500012

[21] Franchignoni F Horak F Godi M Nardone A Giordano A Using psychometric techniques to improve the Balance Evaluation Systems Test: the mini-BESTest J Rehabil Med 2010 42 04 323 331 20461334 10.2340/16501977-0537 PMC3228839

[22] Stebbins G T Goetz C G Burn D J Jankovic J Khoo T K Tilley B C How to identify tremor dominant and postural instability/gait difficulty groups with the movement disorder society unified Parkinson's disease rating scale: comparison with the unified Parkinson's disease rating scale Mov Disord 2013 28 05 668 670 23408503 10.1002/mds.25383

[23] Li X Xing Y Martin-Bastida A Piccini P Auer D P Patterns of grey matter loss associated with motor subscores in early Parkinson's disease Neuroimage Clin 2017 17 498 504 29201638 10.1016/j.nicl.2017.11.009 PMC5700824

[24] Oeda T Umemura A Tomita S Hayashi R Kohsaka M Sawada H Clinical factors associated with abnormal postures in Parkinson's disease PLoS One 2013 8 09 e73547 24069205 10.1371/journal.pone.0073547 PMC3777935

[25] Ando Y Fujimoto K I Ikeda K Postural Abnormality in Parkinson's Disease: A Large Comparative Study With General Population Mov Disord Clin Pract (Hoboken) 2019 6 03 213 221 30949552 10.1002/mdc3.12723 PMC6417750

[26] Rosario J L Bezerra Diógenes M S Mattei R Leite J R Differences and similarities in postural alterations caused by sadness and depression J Bodyw Mov Ther 2014 18 04 540 544 25440204 10.1016/j.jbmt.2013.12.010

[27] Balzini L Vannucchi L Benvenuti F Clinical characteristics of flexed posture in elderly women J Am Geriatr Soc 2003 51 10 1419 1426 14511162 10.1046/j.1532-5415.2003.51460.x

[28] Gong H Sun L Yang R Changes of upright body posture in the sagittal plane of men and women occurring with aging - a cross sectional study BMC Geriatr 2019 19 01 71 30836933 10.1186/s12877-019-1096-0 PMC6402106

[29] Yoshii F Moriya Y Ohnuki T Ryo M Takahashi W Postural deformities in Parkinson's disease -Mutual relationships among neck flexion, fore-bent, knee-bent and lateral-bent angles and correlations with clinical predictors J Clin Mov Disord 2016 3 1 26835153 10.1186/s40734-016-0029-8 PMC4731916

[30] Miller I N Cronin-Golomb A Gender differences in Parkinson's disease: clinical characteristics and cognition Mov Disord 2010 25 16 2695 2703 20925068 10.1002/mds.23388 PMC3003756

[31] Bartolić A Pirtosek Z Rozman J Ribaric S Postural stability of Parkinson's disease patients is improved by decreasing rigidity Eur J Neurol 2005 12 02 156 159 15679705 10.1111/j.1468-1331.2004.00942.x

[32] Papapetropoulos S Ellul J Argyriou A A Chroni E Lekka N P The effect of depression on motor function and disease severity of Parkinson's disease Clin Neurol Neurosurg 2006 108 05 465 469 16150537 10.1016/j.clineuro.2005.08.002

[33] Hassan A Vallabhajosula S Zahodne L B Correlations of apathy and depression with postural instability in Parkinson disease J Neurol Sci 2014 338(1-2):162 165 24461565 10.1016/j.jns.2013.12.040

[34] Ziropadja Lj Stefanova E Petrovic M Stojkovic T Kostic V S Apathy and depression in Parkinson's disease: the Belgrade PD study report Parkinsonism Relat Disord 2012 18 04 339 342 22166396 10.1016/j.parkreldis.2011.11.020

[35] Michalak J Troje N F Fischer J Vollmar P Heidenreich T Schulte D Embodiment of sadness and depression–gait patterns associated with dysphoric mood Psychosom Med 2009 71 05 580 587 19414617 10.1097/PSY.0b013e3181a2515c

[36] Niedenthal P M Embodying emotion Science 2007 316(5827):1002 1005 17510358 10.1126/science.1136930

[37] Coulson M Attributing Emotion to Static Body Postures: Recognition Accuracy, Confusions, and Viewpoint Dependence J Nonverbal Behav 2004 28 117 139

[38] Reijnders J SAM Scholtissen B Weber W EJ Aalten P Verhey F R Leentjens A F Neuroanatomical correlates of apathy in Parkinson's disease: A magnetic resonance imaging study using voxel-based morphometry Mov Disord 2010 25 14 2318 2325 20669264 10.1002/mds.23268

[39] Beuter A Hernández R Rigal R Modolo J Blanchet P J Postural sway and effect of levodopa in early Parkinson's disease Can J Neurol Sci 2008 35 01 65 68 18380279 10.1017/s0317167100007575

